# *GSK3B* and *MAPT* Polymorphisms Are Associated with Grey Matter and Intracranial Volume in Healthy Individuals

**DOI:** 10.1371/journal.pone.0071750

**Published:** 2013-08-12

**Authors:** Carol Dobson-Stone, Patsie Polly, Mayuresh S. Korgaonkar, Leanne M. Williams, Evian Gordon, Peter R. Schofield, Karen Mather, Nicola J. Armstrong, Wei Wen, Perminder S. Sachdev, John B. J. Kwok

**Affiliations:** 1 Neuroscience Research Australia, Randwick, New South Wales, Australia; 2 Department of Pathology and Inflammation and Infection Research Centre, School of Medical Sciences, University of New South Wales, Kensington, Australia; 3 The Brain Dynamics Centre, University of Sydney Medical School and Westmead Millennium Institute, Westmead, Australia; 4 Brain Resource International Database, Brain Resource Ltd., Ultimo, Sydney, New South Wales, Australia, and San Francisco, California; 5 Euroa Centre, Prince of Wales Hospital, Randwick, Australia; 6 Cancer Program, Garvan Institute of Medical Research, Sydney, Australia, School of Mathematics and Statistics, and Prince of Wales Clinical School, University of New South Wales, Sydney, Australia; 7 School of Psychiatry, University of New South Wales, Sydney, Australia; Thomas Jefferson University, United States of America

## Abstract

The microtubule-associated protein tau gene (*MAPT*) codes for a protein that plays an integral role in stabilisation of microtubules and axonal transport in neurons. As well as its role in susceptibility to neurodegeneration, previous studies have found an association between the *MAPT* haplotype and intracranial volume and regional grey matter volumes in healthy adults. The glycogen synthase kinase-3β gene (*GSK3B*) codes for a serine/threonine kinase that phosphorylates various proteins, including tau, and has also been associated with risk for neurodegenerative disorders and schizophrenia. We examined the effects of *MAPT* and two functional promoter polymorphisms in *GSK3B* (rs3755557 and rs334558) on total grey matter and intracranial volume in three independent cohorts totaling 776 neurologically healthy individuals. *In vitro* analyses revealed a significant effect of rs3755557 on gene expression, and altered binding of at least two transcription factors, Octamer transcription factor 1 (Oct-1) and Pre-B-cell leukemia transcription factor 1 (Pbx-1), to the *GSK3B* promoter. Meta-analysis across the three cohorts revealed a significant effect of rs3755557 on total grey matter volume (summary B = 0.082, 95% confidence interval = 0.037–0.128) and intracranial volume (summary B = 0.113, 95% confidence interval = 0.082–0.144). No significant effect was observed for *MAPT* H1/H2 diplotype or *GSK3B* rs334558 on total grey matter or intracranial volume. Our genetic and biochemical analyses have identified a role for *GSK3B* in brain development, which could have important aetiological implications for neurodegenerative and neurodevelopmental disorders.

## Introduction

Microtubule-associated protein tau is a phosphorylated protein highly expressed in brain, where it assists in stabilisation of the cytoskeleton and axonal transport in neurons [1]. Neurofibrillary tangles of hyperphosphorylated tau are a pathological hallmark of several neurodegenerative disorders, including Alzheimer’s disease and frontotemporal dementia [2]. Mutations in the gene encoding tau (*MAPT*) are found in 9–21% of cases of familial frontotemporal dementia [3], [4]. A large region of linkage disequilibrium covers the gene and is denoted by two major haplotypes, termed H1 and H2 [5]. H1/H2 haplotypes have been shown to affect expression and/or splicing of *MAPT* [6]–[10] and have been associated with risk of several sporadic neurodegenerative disorders, including Alzheimer’s disease [11], progressive supranuclear palsy [12], corticobasal degeneration [13] and Parkinson’s disease [14].

Glycogen synthase kinase-3β (GSK-3β) is a serine/threonine kinase that phosphorylates a variety of nuclear and cytoplasmic proteins, including tau and β-catenin [15], [16]. Polymorphisms in the gene encoding GSK-3β (*GSK3B*) have been associated with Alzheimer’s disease and frontotemporal dementia [17], [18], and an epistatic interaction between *GSK3B* and *MAPT* has been associated with Parkinson’s disease and Alzheimer’s disease [9], [19], [20]. As well as neurodegeneration, GSK-3β plays a key role in neurodevelopment [21] and *GSK3B* polymorphisms have been associated with schizophrenia [22]. *In vitro* studies have indicated that a number of polymorphisms have a functional effect on *GSK3B* gene expression and/or splicing [20], [22], including rs334558, which lies 18 bp upstream of *GSK3B* exon 1. The minor allele of rs334558 (in European populations) [23] is predicted to abrogate binding of transcription factor AP4 and was associated with a decrease in gene expression relative to the major allele [20].

As well as a role in susceptibility to neurodegeneration, recent studies have implicated polymorphisms in the *MAPT* region as implicated in brain structure and development in healthy individuals. A voxel-based morphometry (VBM) study found reduced grey matter (GM) volumes in several brain regions in H1 carriers [24]. Subsequently, a genome-wide association study found significant association between intracranial volume (ICV), a measurement reflecting lifetime maximal brain size, and H1/H2 diplotype [25]. Given GSK-3β’s role in phosphorylation of β-catenin, whose overexpression leads to grossly increased cerebral cortical size [26], [27], *GSK3B* is an excellent candidate gene for influencing brain size parameters. Here we examined the effects of two functional single nucleotide polymorphisms (SNPs) located in the promoter region of *GSK3B* (rs3755557 and rs334558), and the H1/H2 haplotype of *MAPT* on grey matter and intracranial volume in three cohorts of neurologically healthy individuals.

## Materials and Methods

### Ethics Statements

The procedures in this study were approved by the Human Research Ethics Committees of the University of New South Wales, the South Eastern Sydney and Illawarra Area Health Service, the Sydney West Area Health Service, the ethics committee of the Australian Twin Registry, University of Melbourne and Queensland Institute of Medical Research. All participants gave written informed consent.

### Subjects

Three groups of healthy individuals of European ancestry were examined in this study, derived from the following cohorts: the Brain Resource International Database (BRID); the Sydney Memory and Aging Study (MAS); and the Older Australian Twin Study (OATS). Demographic details for these cohorts are provided in Table 1.

**Table 1:** Demographics of cohorts examined in this study.

			*MAPT* diplotype^b^			*GSK3B* rs3755557b,c			*GSK3B* rs334558b,d		
Cohort	N (M/F)	Age (y)^a^	H1H1	H1H2	H2H2	TT	AT	AA	AA	AG	GG
BRID	87 (47/40)	41.6±17.0	58 (66.7)	26 (29.9)	3 (3.4)	59 (67.8)	28 (32.2)	0 (0.0)	35 (40.2)	42 (48.3)	10 (11.5)
MAS	489 (217/272)	78.4±4.7	287 (58.7)	169 (34.6)	33 (6.7)	355 (72.6)	120 (24.5)	14 (2.9)	200 (40.9)	221 (45.2)	68 (13.9)
OATS	200 (81/119)	70.5±5.1	124 (62.0)	65 (32.5)	11 (5.5)	143 (71.5)	53 (26.5)	4 (2.0)	91 (45.5)	80 (40.0)	29 (14.5)

a Mean ± standard deviation.

b Genotype counts (percentages in parentheses).

c Genotype counts for MAS and OATS cohorts derived from imputed allele dosage scores, rounded to the nearest whole number. Raw dosage scores were used for linear regression analyses.

d Genotype counts for OATS cohort derived from imputed allele dosage scores, rounded to the nearest whole number. Raw dosage scores were used for linear regression analyses.

The BRID cohort is a cross-sectional database of healthy individuals with extensive neuropsychological and brain imaging data (http://www.brainresource.com) [28]. Caucasian volunteers (n = 363) were from the Brain Resource International Database, governed for scientific purposes by the Brain Research And Integrative Neuroscience Network (BRAINnet). Informed written consent was provided in accordance with local human research ethical requirements. Participants were excluded if they demonstrated a family history of a genetic disorder or a personal history of mental illness, drug or alcohol addiction, physical brain injury, neurological disorder or other serious medical condition. Additional inclusion criteria for this study were availability of DNA for genotyping, availability of Magnetic Resonance Imaging (MRI) data and age of 20 years or over. This age cutoff was used in this relatively young cohort to avoid inclusion of participants who had not yet reached maximal brain volume. MRI was performed on 1.5-T Siemens Vision Plus and Siemens Sonata systems (Siemens, Erlangen, Germany). T1-weighted MRI acquisition and analysis was performed as described previously [29].

The Sydney MAS cohort is a longitudinal study of non-demented, community-dwelling individuals aged 70–90 years old at baseline. MAS participants were recruited randomly from areas of Eastern Sydney, Australia via the electoral roll, for which registration is compulsory. Individuals were excluded if they had an adjusted Mini-Mental State Examination score <24 [30], a diagnosis of dementia, mental retardation, psychotic disorder (including schizophrenia and bipolar disorder), multiple sclerosis, motor neuron disease, progressive malignancy, or inadequate English to complete assessments. Details of the sampling methodology have been published previously [31]. All 1037 MAS participants were administered a comprehensive neuropsychological test battery at baseline (for details see [31]). Of these participants, 542 (52.3%) also had T1-weighted structural MRI scans as previously described [31]. Volumes for specific brain regions were derived from atlas-based parcellation. [31].

For the OATS cohort, twins were recruited through the Australian Twin Registry as well as by a new recruitment drive by the authors. The inclusion criteria were age 65 years and older, residence in New South Wales, Victoria or Queensland, ability to consent, having a consenting co-twin, and having completed some education in English to be able to complete the questionnaires and neuropsychological testing in English. The exclusion criteria included: current diagnosis of life-threatening medical illness, intellectual handicap, or acute psychotic disorder. One twin per pair was selected at random for association analyses. MRI data were obtained on three 1.5 Tesla and one 3 Tesla scanners at three imaging centres as described in Batouli et al. [32].

Intracranial volumes for all three cohorts were calculated by summation of total grey matter, white matter and cerebrospinal fluid volumes.

### Luciferase Reporter Gene Assay

A 1795 bp DNA fragment comprising the promoter and transcription start site of *GSK3B* was PCR amplified using the primers GSKProm3F 5′-TCAAAGCAAGAGCCAGGTAATCTG –3′ and GSKProm2R from genomic DNAs of individuals with representative genotypes for the two *GSK3B* SNPs, and subcloned into the pGL3-Basic Luciferase vector (Promega, Madison, WI, USA). Each promoter haplotype was assayed for transcriptional efficiency. Each recombinant vector was transfected as triplicate wells into human embryonic kidney 293 cells (ATCC CRL 1573) using Lipofectamine 2000 according to manufacturer’s instructions (Invitrogen, Carlsbad, CA, USA). Cells were harvested after 48 hours and cell lysates were assayed for luciferase activity using the Bright-Glo Luciferase assay system (Promega). The experiment was performed independently three times.

### Electrophoretic Mobility Shift Assay

32-mers were designed for each allele of rs3755557 as follows: the ‘A’ allele using the primers 5′ -CCAGAAAGCACATGTAAAAGGACCTATATTTG-3′ and 3′ –GGTCTTTCGTGTACATTTTCCTGGATATAAAC-5′; and the ‘A’ allele using the primers 5′ -CCAGAAAGCACATGTTAAAGGACCTATATTTG-3′ and 3′ –GGTCTTTCGTGTACAATTTCCTGGATATAAAC-5′. Octamer transcription factor-1 (Oct-1) and Pre-B-cell leukemia transcription factor 1 (Pbx-1) proteins were generated by *in vitro* coupled transcription-translation reactions using the TNT T7 rabbit reticulocyte lysate system (Promega). A double stranded oligonucleotide fragment from each allele was end-labelled using T4 polynucleotide kinase and [γ-^32^P]ATP. DNA-protein binding reactions containing 20 µl of 10 mM HEPES (pH 7.9), 1 mM dithiothreitol, 1 µg/µl poly (dI-dC), 10% glycerol, 2 ng of labelled DNA oligonucleotide fragments and *in vitro* translated proteins were incubated for 20 minutes at room temperature. DNA-protein complexes were electrophoresed through a 6% nondenaturing polyacrylamide gel in 0.5x Tris-borate EDTA and visualised by autoradiography.

### Direct Genotyping of Single Nucleotide Polymorphisms

A TaqMan Probe Genotyping Assay (ABI Biosystems, Foster City, CA) for SNP rs1052553 was used to determine the corresponding H1/H2 *MAPT* diplotype in the BRID cohort. The G allele of rs1052553 corresponds to the H2 haplotype. rs242559, previously genotyped as part of the Affymetrix SNP 6.0 array (Affymetrix Inc, Santa Clara, CA), was used to determine the corresponding H1/H2 diplotype in the MAS cohort. The C allele of rs242559 corresponds to the H2 haplotype. For the OATS cohort, rs1052553, which was previously genotyped on the Illumina Express SNP chip (Illumina Inc, San Diego, CA), was used.

The *GSK3B* SNP rs334558 was genotyped in the MAS cohort by means of a TaqMan Probe Genotyping Assay. SNPs rs3755557 & rs334558 were genotyped in the BRID cohort by restriction length fragment polymorphism analysis. Polymorphisms were amplified using the following primers: rs3755557 using GSKProm1F 5′-GCCGCCATCCTGATTGTAATCCAGTGG-3′ and GSKProm1R 5′-GCTTACTTTGTTCTGTCCCAAGTCC-3′; rs334558 using GSKProm2F 5′-TTTATAGACGCCCTCCCTTCGCTT-3′ and GSKProm2R 5′-TTCCTTCCTTCCTTTGTCACTTGGC-3′. Each SNP was detected by restriction length fragment polymorphism using the following restriction enzymes: *Mse* I (New England Biolabs, Beverly, MA, USA), which cleaves the T allele of rs3755557; and *Alu* I (New England Biolabs), which cleaves the A allele of rs334558.

### Imputation of Additional Polymorphisms

rs3755557 was imputed for the MAS cohort and rs3755557 & rs334558 were imputed for the OATS cohort using the 1000 Genome Project phased haplotype data Imputation for both cohorts was carried out using MaCH/minimac according to the ENIGMA protocols (http://enigma.loni.ucla.edu) [33], [34].

### Statistical Analysis

Primary statistical analyses were performed using IBM SPSS Statistics v20. Luciferase expression levels were compared by Student’s *t* test (two-tailed). For the electrophoretic mobility shift assay, band intensities were compared using a paired two-tailed *t* test. Differences were considered significant at *p*<0.05. The effects of polymorphisms on total grey matter volume, intracranial volume and regional grey matter volumes were examined by linear regression analysis. Age and sex were included as *a priori* predictor variables for total grey matter and intracranial volume analysis; age, sex and intracranial volume were used for analysis of regional grey matter volumes. Meta-analysis was performed by conversion of grey matter and intracranial volumes to Z scores for each cohort, followed by combining unstandardised coefficients and standard errors according to the procedure described by Neyeloff et al [35], using a random effects model. Meta-analyses were considered significant at *p*<0.0083, corresponding to *p*<0.05 adjusted for six comparisons.

## Results

### Analysis of rs3755557 Effect on Promoter Function

For this study, we defined the minor alleles of rs3755557 and rs334558 according to the nucleotide sequence on the plus strand of chromosome 3 as to be consistent with the SNPs annotations in public databases [23], i.e. the opposite orientation as the *GSK3B* gene, so that the minor allele for rs3755557 = A and rs334558 = G. We have previously shown that the rs334558 SNP modulates transcriptional efficiency of the *GSK3B* promoter [20]. We examined the promoter sequence of *GSK3B* for additional possible binding sites for transcription factors using the MatInspector v2.2 software and the TRANSFAC 4.0 database [36], using a high stringency of selection (maximal ‘Core similarity’ setting of 1 and ‘Matrix similarity’ of 0.85). The polymorphism rs3755557 is located 1693 bp upstream of *GSK3B* exon 1, within putative binding sites for developmentally important transcription factors such as Oct-1 and Pbx-1.

**Figure 1:**
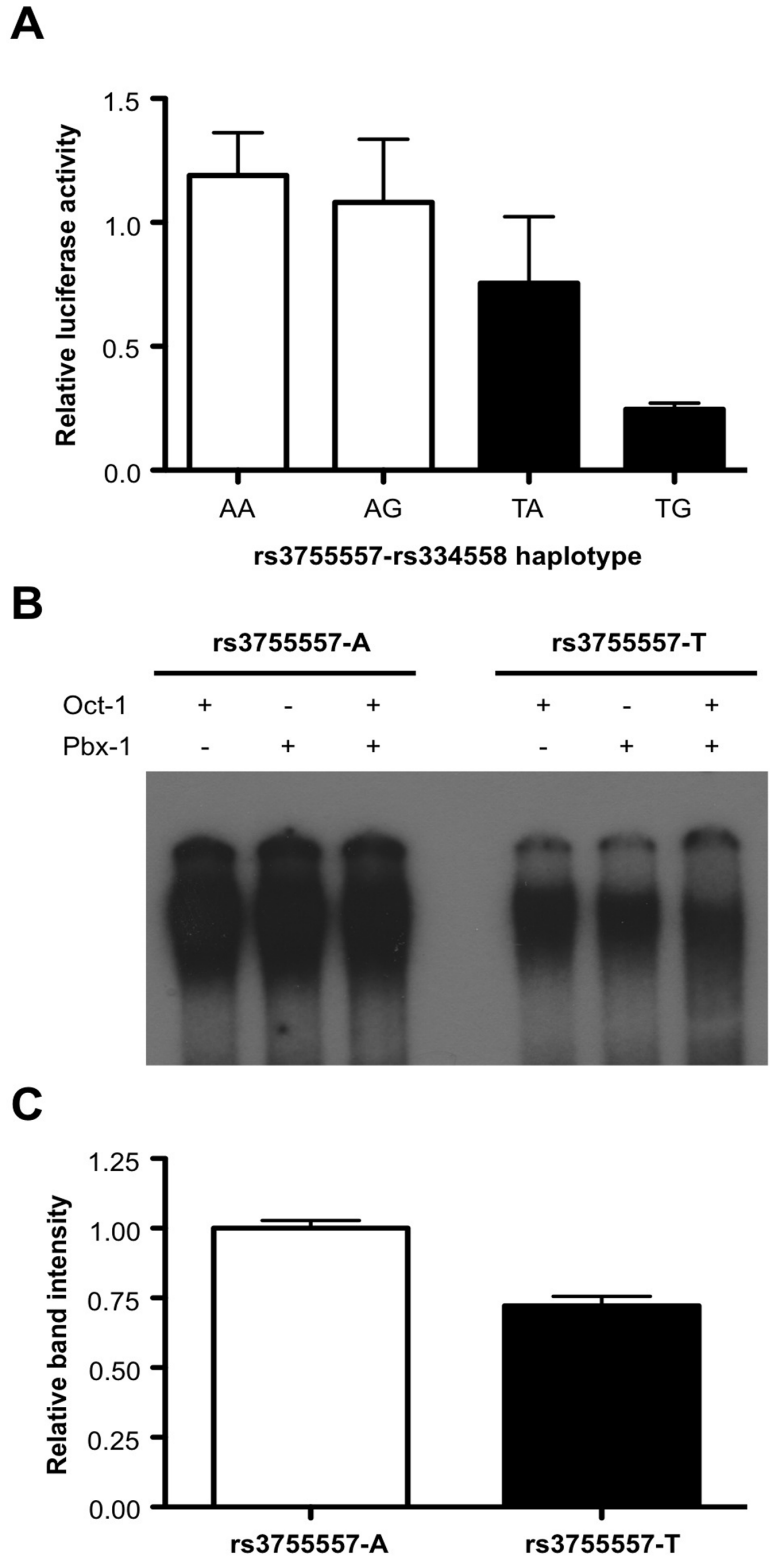
Effect of *GSK3B* rs3755557 on transcription. a) Comparison of expression levels from *GSK3B* promoter constructs by luciferase reporter gene assay. Constructs contained either the A or T allele of rs3755557 on a background of either the T or C allele of rs334558. Error bars indicate standard error of the mean from 3 independent experiments. *, *p*<0.05. b) Electrophoretic mobility shift assay to show differential binding of either Oct-1 or Pbx-1 transcription factors to labeled oligomers corresponding to either the A or T allele of rs3755557. c) Quantification of gel shift assay. Error bars indicate standard error of the mean density of bands corresponding to (Oct-1), (Pbx-1) and (Oct-1+ Pbx-1) binding for each allele.

The T allele was predicted to abrogate binding of transcription factors to this site. We therefore examined the ability of rs3755557 to affect the expression of a luciferase reporter gene. As shown in Figure 1A, mean expression from the T allele promoter constructs were significantly lower than those from the A allele (0.4 fold expression relative to the A allele, *t*_(10)_ = 2.92, *p* = 0.015). We also performed a electrophoretic mobility shift assay using labelled oligomers corresponding to the A or T alleles of rs3755557 (Figure 1B and C, Figure S1 in Information S1). The T allele was associated with weaker binding to transcription factors Oct-1 and Pbx-1 than the T allele (0.7 fold binding relative to the A allele, *t*_(2)_ = 6.12, *p* = 0.026), consistent with its weaker promoter activity in the luciferase assay.

### Association Analysis with Total Grey Matter and Intracranial Volume

We examined whether grey matter volume or intracranial volume differed significantly by *MAPT* H1/H2 diplotype or *GSK3B* genotypes in three MRI cohorts by linear regression analysis (Table 2, Table S1 in Information S1 and Table S2 in Information S1). Age and sex were included as *a priori* predictors. *MAPT* diplotype was a significant predictor of total grey matter in the MAS cohort only (*p* = 0.004), with the H1 haplotype predicting lower grey matter volume. *MAPT* diplotype was not a significant predictor of intracranial volume in any individual cohort. We detected a non-significant trend effect of *GSK3B* rs3755557 genotype on total grey matter in the OATS cohort (*p* = 0.077), and on intracranial volume in the BRID (*p* = 0.098) and OATS cohorts (*p* = 0.074). In each case, the A allele predicted lower grey matter or intracranial volume. *GSK3B* rs334558 was not a significant predictor of total grey matter or intracranial volume in any individual cohort.

**Table 2:** Linear regression analyses to assess effect of *MAPT* diplotype and *GSK3B* genotypes on total grey matter and intracranial volume^a^.

Phenotype	Predictorvariable^b^	Cohort									Meta-analysis B (95% CI)^c^
		BRID			MAS			OATS			
		β	*t*	*p*	β	*t*	*p*	β	*t*	*p*	
Total grey matter	*MAPT* H1/H2	0.010	0.13	0.898	0.111	2.92	0.004	0.025	0.44	0.657	0.121 (0.022–0.221)
	*GSK3B* rs3755557	0.095	1.24	0.218	0.036	0.94	0.348	0.100	1.78	0.077	**0.082 (0.037–0.128)**
	*GSK3B* rs334558	0.112	1.50	0.138	0.033	0.87	0.387	0.060	1.05	0.294	0.041 (−105.0–105.0)
Intracranial volume	*MAPT* H1/H2	0.018	0.22	0.828	0.062	1.57	0.118	0.025	0.45	0.653	0.015 (−0.057–0.087)
	*GSK3B* rs3755557	0.136	1.67	0.098	0.056	1.41	0.158	0.097	1.79	0.074	**0.113 (0.082–0.144)**
	*GSK3B* rs334558	0.102	1.27	0.207	0.020	0.49	0.622	0.056	1.030	0.304	0.029 (−658.2–658.2)

a Genotypes were tested separately in models including age and sex as *a priori* predictors. Values for all predictors in each model are given in Tables S1 and S2 in Information S1.

b Coded as follows: *MAPT*, H1H1 = 0, H1H2 = 1, H2H2 = 2; rs3755557, AA = 0, AT = 1, TT = 2; rs334558, GG = 0, AG = 1, AA = 2.

c Meta-analyses with a significant effect after correction for multiple comparisons are in **bold.**

We performed meta-analyses to determine whether there was an overall effect of *MAPT* diplotype or *GSK3B* SNPs on grey matter or intracranial volume across the three cohorts. *GSK3B* rs3755557 genotype was a significant predictor of both total grey matter (summary B = 0.082, 95% confidence interval = 0.037–0.128) and intracranial volume (summary B = 0.113, 95% confidence interval = 0.082–0.144). Neither *MAPT* nor *GSK3B* rs334558 were significant predictors of total grey matter or intracranial volume by meta-analysis. Note that we have calculated the I^2^ value, which describes the percentage of variability due to heterogeneity [35] for each meta-analysis performed in this study. The I^2^ values exceed the consensus threshold of 50% for all meta-analyses with the exception of the *MAPT* diplotype and grey matter volume (I^2^ = 6.2), and thus warrant the use of the random effects form of meta-analysis. It should be noted that the p value does reach signficance (p<0.0083) for *MAPT* diplotype and grey matter volume when analysed using either the fix or random effects form of meta-analyses. For consistency, we have presented only the data from the conservative random-effects analyses.

### Analysis of *MAPT* H1/H2 Diplotype and Regional Grey Matter Volumes

Regional grey matter volumetric data was available for the MAS cohort. We examined whether *MAPT* diplotype was associated with grey matter volume differences in candidate brain regions (right orbital frontal cortex, left insula, right caudate, right inferior temporal gyrus, right inferior lobe of cerebellum (Crus II)) previously identified by Canu et al [24] (Table 3). Linear regression analysis using age, sex and intracranial volume as *a priori* predictors revealed that *MAPT* diplotype was a predictor of left insula and right cerebellar Crus II lobar volumes at a nominally significant level (*p* = 0.039 and *p* = 0.033, respectively), with the H1 haplotype predicting lower grey matter volumes. However, these findings were not significant when corrected for multiple comparisons for five regions (i.e., *p*>0.01).

**Table 3:** Linear regression analyses to assess effect of *MAPT* diplotype on regional brain volumes^a^.

Region	*MAPT* H1/H2^b^		
	β	*t*	*p*
Right orbital frontal cortex	0.040	1.15	0.250
Left insula	0.074	2.07	0.039
Right caudate	−0.025	−0.65	0.519
Right inferior temporal gyrus	0.048	1.20	0.231
Right cerebellum, Crus II	0.089	2.13	0.033

a Models included age, sex and intracranial volume as *a priori* predictors.

b Coded as follows: H1H1 = 0, H1H2 = 1, H2H2 = 2.

## Discussion

This study has demonstrated that rs3755557, a SNP in the promoter region of *GSK3B*, modulates gene expression by altered binding of transcription factors and is significantly associated with intracranial volume across three cohorts of healthy adults (total n = 776). Our luciferase reporter assay demonstrated that the minor A allele of rs3755557 supported twice the level of the expression levels compared to the major T allele. The electrophoretic mobility shift assay gave a plausible explanation for this enhanced expression, namely the increased binding of transcription factors to the A allele. This increased expression of the A allele and altered binding of transcription machinery is consistent with the findings of an earlier study reported last year [22] who also reported increased transcriptional activity associated with the minor allele of rs3755557. Moreover, we were able to identify two transcription factors with altered binding, namely Oct-1 and Pbx-1.

Both Oct-1 and Pbx-1 have important roles in organ development. Oct-1 (also known as POU2F1) is found in all tissue types [37], and throughout mouse embryogenesis and regulates a large group of target genes [38], [39]. Amongst multiple other roles, Oct-1 has been found to play a role in the development of the central nervous system [40]. Similarly, Pbx-1 regulates developmental gene expression in a variety of tissues, including the central nervous system, and has been implicated in axonal pathfinding [41]. It is possible that part of the role played by Oct-1 and Pbx-1 in brain development is via altering expression of *GSK3B*.

Consistent with this hypothesis, we found a significant effect of *GSK3B* SNP rs3755557 on intracranial volume, a measurement that is largely determined by the maximal brain size reached in development. Carriers of the A allele of rs3755557 are hypothesised to have higher expression of *GSK3B*, which leads to over-phosphorylation of its target proteins, possibly including tau and/or β-catenin, which in turn leads to reduced proliferation of neuronal precursors and thus a smaller brain size. Interestingly, smaller intracranial volume is associated with an increased susceptibility to senile dementia [42], and a recent meta-analysis of over 9000 patients demonstrated a significant association of schizophrenia with reduced intracranial volume [43]. Given that *GSK3B* is implicated in development of both Alzheimer’s disease and schizophrenia [9], [17]–[19], [22], this suggests that at least part of the relationship of these disorders with reduced intracranial volume may reflect the impact of *GSK3B* on brain development.

We did not detect a significant effect of *MAPT* H1/H2 diplotype on total grey matter by meta-analysis, although we did observe a nominally significant effect in our largest cohort. We also did not observe the previously reported association of the *MAPT* linkage disequilibrium region with intracranial volume [25]. Lastly, we were not able to replicate the previously observed regional grey matter volume differences between *MAPT* diplotype groups after correction for multiple comparisons. This may reflect the fact that our analysis was based on comparison of previously parcellated regional grey matter volumes, rather than the voxel-based morphometry analysis in the original study [24]. We note, however, that one of the two regions in our study with a nominally significant effect of *MAPT* was the left insula. This region showed the largest cluster difference in H1H1 versus H2 carriers and the second largest cluster size difference in H2H2 versus H1 carriers in the Canu et al [24] study.

A potential limitation of our study is the size of the individual cohorts used, especially the BRID cohort (n = 87). Although they are relatively large cohorts for imaging studies, individually they lack power to detect genetic effects of small size. We addressed this limitation by performing meta-analysis on genotype-phenotype comparisons that had shown preliminary evidence for association in the primary analyses. The combined cohort (n = 776) had >80% power to detect an effect size corresponding to 1.6% of the variance [44]. Another limitation is that not all SNPs analysed were genotyped directly: rs3755557 and rs334558 were imputed in the MAS cohort, and rs3755557 in the OATS cohort, although the correlation value (r^2^) for these imputed SNPs were all between 0.94 and 0.99. All imputation techniques introduce a margin of error to the genotype calls compared to genotyping the SNPs directly. We did use raw imputed allele dosage scores for each individual to account for the uncertainty in the imputation process but it is possible that the added error in genotype calls may have obscured true effects of genotype on brain size measures. Thirdly, given that the *GSK3B* rs3755557–rs334558 T-G haplotype showed the most marked decrease in gene expression relative to other haplotypes (Fig. 1B), we may have observed a stronger effect on brain size measures had we analysed *GSK3B* 2-SNP haplotypes rather than examining the SNPs separately. Given that at least one of the *GSK3B* SNPs had not been directly genotyped for the two larger cohorts, we felt that estimating haplotypes for these cohorts would introduce an unacceptable level of error to the data that would further obscure any association with phenotypes. In addition, examination of European ancestry populations in the 1000 Genomes project [23] reveals a high degree of linkage disequilibrium between the two SNPs (D′ = 0.96) (Table S3 in Information S1), and the T allele of rs3755557 effectively defines the low-activity T-G haplotype. This may explain why we observed a significant effect of rs3755557 but not rs334558 on intracranial volume.

### Conclusions

In conclusion, we have determined that the minor A allele of rs3755557 leads to increased activity of the *GSK3B* promoter, probably by greater binding of transcription factors such as Oct-1 and Pbx-1, and is associated with reduced intracranial volume and grey matter in healthy adults. This implies a role for *GSK3B* in brain development as well as neurodegeneration, which may have important implications for the treatment of disorders such as Alzheimer’s disease, frontotemporal dementia, Parkinson’s disease and schizophrenia, that have been associated with *GSK3B*.

## Supporting Information

Information S1 (PDF)
