# Supplementary material for: GSK3B and MAPT Polymorphisms Are Associated with Grey Matter and Intracranial Volume in Healthy Individuals
Source: PLoS One. 2013 Aug 12;8(8):e71750. doi: 10.1371/journal.pone.0071750 (PMC3741177; doi:10.1371/journal.pone.0071750)

Supplementary Tables

Supplementary Table S1

Linear regression analyses for total grey matter volume – all predictors

Cohort														
BRID					MAS					OATS				
Model summary	Predictor[a]	β	*t*	*p*	Model summary	Predictor[a]	β	*t*	*p*	Model summary	Predictor[a]	β	*t*	*p*
$F_{(3,86)} = 30.80$,	Age	-0.482	-6.29	$1.4x10^{-8}$	$F_{(3,485)} = 70.85$,	Age	-0.297	-7.83	$3.1x10^{-14}$	$F_{(3,196)} = 37.60$,	Age	-0.009	-0.15	0.878
$p = 1.8x10^{-13}$,	Sex	-0.521	-6.80	$1.4x10^{-9}$	$p = 5.2x10^{-38}$,	Sex	-0.451	-11.90	$7.9x10^{-29}$	$p = 3.1x10^{-19}$,	Sex	-0.603	-10.50	$9.3x10^{-21}$
$R^2_{adj} = 0.510$	*MAPT* H1/H2	0.010	0.13	0.898	$R^2_{adj} = 0.300$	*MAPT* H1/H2	0.111	2.92	0.004	$R^2_{adj} = 0.356$	*MAPT* H1/H2	0.025	0.44	0.657
$F_{(3,86)} = 31.87$,	Age	-0.503	-6.57	$4.1x10^{-9}$	$F_{(3,485)} = 67.25$,	Age	-0.290	-7.58	$1.7x10^{-13}$	$F_{(3,196)} = 39.15$,	Age	-0.011	-0.20	0.845
$p = 8.4x10^{-14}$,	Sex	-0.509	-6.75	$1.9x10^{-9}$	$p = 2.2x10^{-36}$,	Sex	-0.455	-11.92	$6.4x10^{-29}$	$p = 7.2x10^{-20}$,	Sex	-0.608	-10.68	$2.9x10^{-21}$
$R^2_{adj} = 0.519$	*GSK3B* rs3755557	0.095	1.24	0.218	$R^2_{adj} = 0.289$	*GSK3B* rs3755557	0.036	0.94	0.348	$R^2_{adj} = 0.365$	*GSK3B* rs3755557	0.100	1.78	0.077
$F_{(3,86)} = 32.36$,	Age	-0.480	-6.43	$7.8x10^{-9}$	$F_{(3,485)} = 67.19$,	Age	-0.289	-7.56	$2.0x10^{-13}$	$F_{(3,196)} = 38.08$,	Age	-0.008	-0.13	0.895
$p = 5.9x10^{-14}$,	Sex	-0.530	-7.07	$4.4x10^{-10}$	$p = 2.4x10^{-36}$,	Sex	-0.457	-11.97	$4.2x10^{-29}$	$p = 2.0x10^{-19}$,	Sex	-0.606	-10.59	$5.4x10^{-21}$
$R^2_{adj} = 0.522$	*GSK3B* rs334558	0.112	1.50	0.138	$R^2_{adj} = 0.289$	*GSK3B* rs334558	0.033	0.87	0.387	$R^2_{adj} = 0.359$	*GSK3B* rs334558	0.060	1.05	0.294

[a] Coded as follows: sex, male = 0, female = 1; *MAPT*, H1H1 = 0, H1H2 = 1, H2H2 = 2; rs3755557, AA = 0, AT = 1, TT = 2; rs334558, GG = 0, AG = 1, AA = 2

Supplementary Table S2

Linear regression analyses for intracranial volume – all predictors

Cohort														
BRID					MAS					OATS				
Model summary	Predictor[a]	β	*t*	*p*	Model summary	Predictor[a]	β	*t*	*p*	Model summary	Predictor[a]	β	*t*	*p*
$F_{(3,86)} = 23.78$,	Age	0.080	0.98	0.328	$F_{(3,485)} = 52.78$,	Age	-0.062	-1.56	0.119	$F_{(3,196)} = 47.14$,	Age	0.032	0.58	0.565
$p = 3.3x10^{-11}$,	Sex	-0.682	-8.34	$1x10^{-12}$	$p = 1.5x10^{-29}$,	Sex	-0.486	-12.32	$1.6x10^{-30}$	$p = 5.6x10^{-23}$,	Sex	-0.641	-11.66	$3.3x10^{-24}$
$R^2_{adj} = 0.443$	*MAPT* H1/H2	0.018	0.22	0.828	$R^2_{adj} = 0.241$	*MAPT* H1/H2	0.062	1.57	0.118	$R^2_{adj} = 0.410$	*MAPT* H1/H2	0.025	0.45	0.653
$F_{(3,86)} = 25.48$,	Age	0.050	0.62	0.539	$F_{(3,485)} = 52.58$,	Age	-0.059	-1.50	0.136	$F_{(3,196)} = 48.87$,	Age	0.029	0.54	0.590
$p = 8.8x10^{-12}$,	Sex	-0.663	-8.31	$1x10^{-12}$	$p = 1.9x10^{-29}$,	Sex	-0.488	-12.36	$1.1x10^{-30}$	$p = 1.3x10^{-23}$,	Sex	-0.645	-11.85	$9.0x10^{-25}$
$R^2_{adj} = 0.461$	*GSK3B* rs3755557	0.136	1.67	0.098	$R^2_{adj} = 0.241$	*GSK3B* rs3755557	0.056	1.41	0.158	$R^2_{adj} = 0.419$	*GSK3B* rs3755557	0.097	1.79	0.074
$F_{(3,86)} = 24.75$,	Age	0.081	1.01	0.314	$F_{(3,485)} = 51.81$,	Age	-0.057	-1.44	0.150	$F_{(3,196)} = 47.63$,	Age	0.033	0.60	0.551
$p = 1.6x10^{-11}$,	Sex	-0.688	-8.58	$4.5x10^{-13}$	$p = 4.6x10^{-29}$,	Sex	-0.490	-12.37	$9.4x10^{-31}$	$p = 3.6x10^{-23}$,	Sex	-0.643	-11.75	$1.8x10^{-24}$
$R^2_{adj} = 0.472$	*GSK3B* rs334558	0.102	1.27	0.207	$R^2_{adj} = 0.238$	*GSK3B* rs334558	0.020	0.49	0.622	$R^2_{adj} = 0.413$	*GSK3B* rs334558	0.056	1.03	0.304

[a] Coded as follows: sex, male = 0, female = 1; *MAPT*, H1H1 = 0, H1H2 = 1, H2H2 = 2; rs3755557, AA = 0, AT = 1, TT = 2; rs334558, GG = 0, AG = 1, AA = 2

Supplementary Table S3

GSK3B haplotype frequencies in CEU and GBR populations of 1000 Genomes project

rs3755557-rs334558 haplotype	CEU + GBR frequency
T-A	0.649
T-G	0.196
A-G	0.152
A-A	0.004

Haplotype frequencies were calculated with Haploview v4.2, using genotype data from CEU and GBR populations accessed via the 1000 Genomes Browser (http://browser.1000genomes.org/index.html).

Supplementary Figure S1

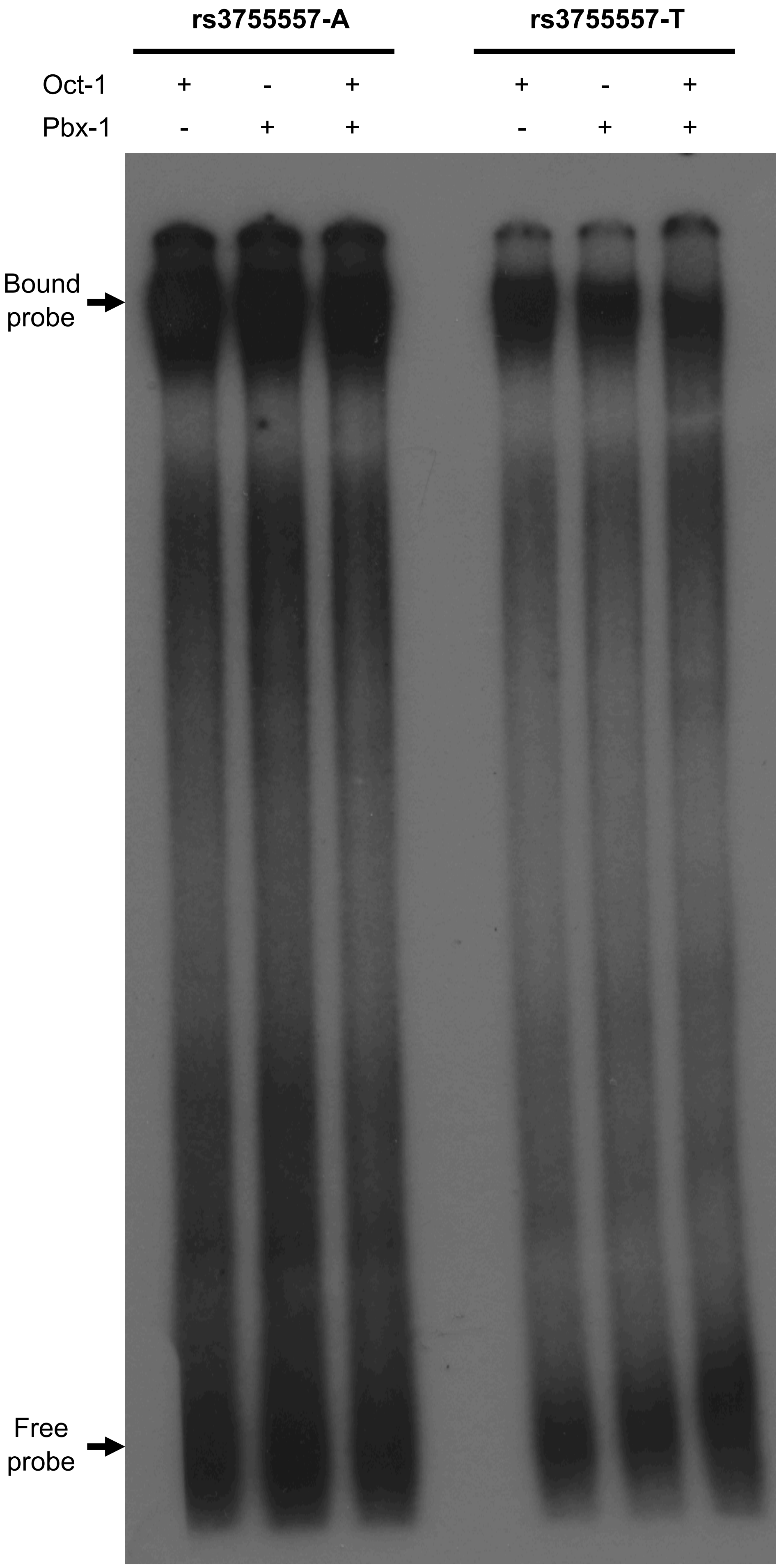

Supplement: Information S1 — (PDF) [file pone.0071750.s001.pdf]
